# Assessing civility at an academic health science center: Implications for employee satisfaction and well-being

**DOI:** 10.1371/journal.pone.0247715

**Published:** 2021-02-26

**Authors:** Lisa A. Campbell, Jenna R. LaFreniere, Mhd Hasan Almekdash, David D. Perlmutter, Huaxin Song, Patricia J. Kelly, Rohali Keesari, Kay Leigh Shannon

**Affiliations:** 1 School of Nursing, Texas Tech University Health Sciences Center, Lubbock, Texas, United States of America; 2 Department of Communication Studies, College of Media & Communication, Texas Tech University, Lubbock, Texas, United States of America; 3 Clinical Research Institute and Department of Surgery, Texas Tech University Health Sciences Center, Lubbock, Texas, United States of America; 4 College of Media & Communication, Texas Tech University, Lubbock, Texas, United States of America; 5 College of Nursing, Thomas Jefferson University, Philadelphia, Pennsylvania, United States of America; 6 Clinical Research Institute, Texas Tech University Health Sciences Center, Lubbock, Texas, United States of America; Peking University, CHINA

## Abstract

Incivilities are pervasive among workers in healthcare institutions. Previously identified effects include deterioration of employee physical and mental health, absenteeism, burnout, and turnover, as well as reduced patient safety and quality of care. This study documented factors related to organizational civility at an academic health sciences center (AHSC) as the basis for future intervention work. We used a cross-sectional research design to conduct an online survey at four of five campuses of an AHSC. Using the Organizational Civility Scale (OCS), we assessed differences across gender, race (White and non-White) and job type (faculty or staff) in the eleven subscales (frequency of incivility, perceptions of organizational climate, existence of civility resources, importance of civility resources, feelings about current employment, employee satisfaction, sources of stress, coping strategies, overall levels of stress/coping ability, and overall civility rating). Significant gender differences were found in six of the eleven subscales: perception of organizational climate (*p* < .001), existence of civility resources (*p* = .001), importance of civility resources (*p* < .001), frequency of incivilities (*p* < .001), employee satisfaction (*p* = .002), and overall civility rating (*p* = .007). Significant differences between respondents by self-identified race were found only in one subscale: existence of civility resources (*p* = .048). Significant differences were found between faculty and staff in four subscales: perception of organizational climate (*p* = .001), importance of civility resources (*p* = .02), employee satisfaction (*p* = .01), and overall levels of stress (*p* = .03). Results suggest that gender and employment type differences exist in the perception of organizational climate at the academic health center, while significant racial differences only occurred in reference to reported existence of civility resources. Attention to these differences should be incorporated into the development of programs to address the problem.

## Introduction

Structural and economic conditions are important determinants of health [1, 2], with employed U.S. adults spending approximately half of their waking hours working [3, 4]. Unfortunately, workplace incivilities are common and have a negative impact on the overall culture, organizational costs, and on the approximately 9.3 million employees reporting such incivilities [5, 6]. Alternatively, affirmative policies and procedures, workforce training, and awareness campaigns have the potential for an ameliorative impact on employee health and well-being [7–9].

As a term, *incivility* can be challenging to define and test because of the broadness of what may be seen by different parties as uncivil versus acceptable discourse [10]. One less politicalized definition is that incivility comprises "rude or disruptive behaviors which often result in psychological or physiological distress for the people involved…" [11 p536]. Incivilities are pervasive in healthcare institutions [12–14]. Uncivil encounters can have specific effects on employees’ physical and mental health [13, 15–19], absenteeism and sick leave [5, 15, 16, 20], burnout and turnover [8], and patient safety, satisfaction, and quality of care [21–29]. The impacts of incivility in the workplace are far-reaching, infusing toxicity at the organizational and individual level, and working their way into patient care. They have a negative effect on organizations, aggregates, and individuals.

Uncivil words or actions may trigger a defensive effect, that is, make people more defensive and less likely to be trusting and collaborative–both crucial requirements for a functional workplace [30]. Employees who view work as part of their identity and are invested in their jobs are even more likely to engage in counterproductive work behaviors and withdrawal when faced with incivility [31]. Examples of such counter-productivity are noted by scholars; "behaviors such as taking long breaks, coming to work late, or purposely doing work slowly may have enabled [targets] to cope with incivility by allowing them to avoid exposure to incivility or retaliate in response to this stressor" [31 p210]. The available research suggests that eliminating incivility is essential in maintaining a healthy workplace culture and retaining invested employees who identify with their jobs.

Although prior studies in healthcare and academic health sciences centers have characterized the experiences of faculty, students, and healthcare professionals (e.g., nurses or physicians), minimal research has examined incivility experiences among staff in these settings [13, 32–34]. Conducting civility research among all employees where health education commences (i.e., an academic health sciences center [AHSC]), is vital because of the large, diverse population with multiple hierarchy layers. Data can provide an understanding of what healthcare workers are practicing and modeling about civility. Based on this reasoning, our study’s purpose was first to characterize incivility and related factors experienced by faculty and staff at a large AHSC, and second, to understand gender, racial, and staff level differences about incivility-associated factors.

## Materials and methods

### Design and sample

We used a cross-sectional research design to conduct an online survey at four of the five campuses of a large AHSC. The sample included staff and faculty in medical, nursing, pharmacy, and health professions schools, a graduate school in biomedical sciences, outpatient clinics, and all university ancillary departments. The Texas Tech University Health Sciences Center Institutional Review Board (IRB) provided approval to conduct the study (#L19-049). The fifth campus operates under a separate review board and, thus, was not included in this study. Respondents in the study were AHSC employees who were between the ages of 18 and 89. Respondents consented to participate by proceeding with the online survey. The survey landing page included the following statement, "By completing the survey, you are giving your consent to be in this research study".

### Measures

*Demographic information* collected included age, gender (male/female/other), race (White/non-White), education level, and job type (staff/faculty). *Civility* was measured with the 88-item Organizational Civility Scale (OCS), which measures the continuum of professional and unprofessional behaviors experienced by employees. The authors signed a copyright licensing agreement with the instrument developers and thus cannot share the instrument (Contact: Boise State University, Office of Technology Transfer, 1910 University Blvd, Boise, ID 83725–2095). The OCS is a widely used instrument with strong reliability and validated measures [35]. Principle Component Analysis (PCA) and Cronbach’s alpha results from our sample for the eleven subscales were analyzed (see Table 1), with findings similar to those of Clark et al.’s [35], ensuring validity and reliability of the instrument. The eleven subscales of the OSC assess:

- frequency of incivility,
- overall civility rating,
- perceptions of organizational climate,
- importance of civility resources,
- existence of civility resources,
- feelings about current employment,
- employee satisfaction,
- sources of stress,
- coping strategies, and
- overall levels of stress and overall coping ability.

**Table 1:** Principle component analysis and Cronbach’s alpha results.

Section#	Section Title	Items Retained	P.C. Extracted	% of Variance Explained	Cronbach ’s α
1	Perception of Organizational Climate	Q4_1, Q4_3, Q4_4, Q4_6, Q4_8, Q4_9, Q4_11, Q4_13, Q4_15, Q4_16	Supervisory Relationships and Values	70.9%	0.95
		Q4_3, Q4_5, Q4_7, Q4_10, Q4_12, Q4_14	Co-worker Relationships		0.91
2A	Ratings of Civility Resources (Existence)	Q5_5, Q5_6, Q5_7, Q5_8, Q5_9, Q5_10, Q6_1, Q6_2, Q6_3, Q6_4, Q6_5, Q6_6, Q6_7, Q6_8, Q6_9	Procedures and Mechanisms for Dealing with Incivility	69.1%	0.96
		Q5_1, Q5_2, Q5_3, Q5_4, Q5_6	Policy Documentation		0.90
2B	Ratings of Civility Resources (Importance)	Q8_1, Q8_2, Q8_3, Q8_4, Q8_5, Q8_6, Q8_7, Q8_8, Q8_9, Q8_10, Q8_11, Q8_12	Infrastructure for Increasing Civility/ Decreasing Incivility	77.2%	0.97
		Q7_1, Q7_2, Q7_3, Q7_4, Q7_5, Q7_6, Q7_7	Written Procedures (Policy Documentation)		0.94
3	Frequency of Incivility	Q29_1……. Q29_16	NFE^†^	NA	0.96
4	Feelings about Current Employment	Q11_1……. Q11_12	NFE: items do not comprise a subscale	NA	0.37
5	Employee Satisfaction	Q12_1…….. Q12_6	NFE†	NA	0.87
6	Sources of Stress	Q13_1…….. Q13_5	NFE†	NA	0.79
7	Coping Strategies	Q14_2, Q14_4, Q14_5	Passive Coping (Avoidance)	53.6%	0.67
		Q14_1, Q14_3, Q14_6	Active Coping		0.34
8A	Overall Levels of Stress	Q15_1	No factors: Only one item section	NA	NA
8B	Overall Levels of Coping Ability	Q16_1	No factors: Only one item section	NA	NA
9	Overall Civility Ratings	Q17_1……. Q17_4	NFE†	NA	0.85

^†^: No Factors Emerged (NFE): unidimensional.

Responses used a Likert-type agreement scale (e.g., *strongly disagree* to *strongly agree* or *completely untrue* to *completely true*) [35].

### Sample size and power analysis

Sample size estimation for this study was conducted in two steps 1) to identify a representative sample for the surveyed population and 2) to decide the minimum number of participants required for the statistical tests to detect true effects and sufficient power. Assuming that the total number of employees in the surveyed institution is 5,000, calculations for a representative sample of the population for survey purposes showed that the minimum required *N* is 357 participants assuming a 95% confidence level and 5% margin of error. Since the initial purpose of this study was to compare groups after conducting the appropriate missing data imputation and validity and reliability testing using PCA and Cronbach’s alpha tests, a priori power analysis was conducted to decide the minimum sample size needed for the main hypothesis testing statistical procedures to detect true effects. Power analysis showed that the minimum sample size required was 156 (assuming that effect size partial η^2^ = 0.06, α = 0.05, power = 80%, and number of groups = 3). The power calculations were conducted for parametric tests. Still, since we conducted the non-parametric alternative due to the ordinal nature of the survey items and test assumptions, the final minimum sample size calculated was multiplied by a correction factor, the asymptotic relative efficiency (ARE) of 0.95 [36]. The minimum sample size for the non-parametric test to detect true effects is approximately 149, considering the correction factor and assuming a parent Gaussian distribution of the examined data. Our sample size of 1,043 in this study meets and exceeds the minimum numbers required for a representative sample of the population for survey purposes and to achieve 80% power.

### Data collection

The survey was disseminated electronically to 6,264 health sciences center employees’ (non-salaried and salaried faculty and staff) work emails by the information services department over a six-week period in Spring 2019. Employee (full-time equivalent = 0) baseline (*N* = 4,956) demographics for the month preceding the survey launch were collected for comparison. Data was collected through an online survey formatted in Qualtrics Software, Version January 2019 (Qualtrics, Provo, UT). Elements of the Dillman method [37] shown to increase participation in surveys guided recruitment. Employees were sent a survey pre-launch notification one week before the launch, a survey launch notification, and a reminder one week before the survey closed. Respondents were informed in each email message that "Your director, supervisor, or manager will not have access to the results of your individual survey".

### Data analysis

We corrected for missing values with Multivariate Imputations by Chained Equations (MICE) in R statistical programming language using the "mice" package as described by Van Buuren and Groothuis-Oudshoorn [38]. Respondents who did not complete the survey beyond demographic items were dropped from the analysis to accurately estimate missing data points. We analyzed subscale score differences across categorical demographic variables (gender, race, and job type) using the Mann-Whitney U test and Kruskal-Wallis with Bonferroni-adjusted Dunn’s post hoc test for pairwise comparisons where appropriate. A *p-value* of less than.05 was set as statistically significant. Hypothesis-testing statistical analyses were performed with IBM SPSS version 25.

## Results

Of the 1,560 employees (24.90%) who responded, 16.65% were dropped because of multiple missing cells, resulting in a final sample size of 1,043. The majority of respondents were White (79.96%) female (76.61%), staff (82.83%), aged 41–55 years (55.99%), with more than five years of service (56.95%); 22.53% had some college and 37.39% a graduate degree. When compared to the AHSC baseline demographics, there was a higher response rate for females, *X*^*2*^ (1, *n* = 799) = 20.50, *p* < .001 than males, Whites, *X*^*2*^ (1, *n* = 832) = 93.41, *p* < .001 as compared to non-Whites, staff, *X*^2^ (1, *n* = 864) = 5.61, *p* = .018 than faculty, those aged 41–55, *X*^2^ (1, *n* = 584) = 178.46, *p* < .001 as compared to the other two age groups, and those who worked in the school of nursing, *X*^*2*^ (1, *n* = 74) = 94.19, *p* < .001 as compared to all other areas of work (see Table 2).

**Table 2:** Demographics of respondents (sample) & academic health sciences center (population).

Characteristics	Respondents *n* = 1043 (%)	AHSC *n* = 4956 (%)	χ^2^, P- value
Gender			
*Female*	799 (76.61)	3558 (71.79)	20.50, < .001
*Male*	216 (20.71)	1398 (28.21)	
*Did not report/describe*	28 (2.68)	N.A.	
Race			
*White*	832 (79.76)	3186 (64.29)	93.41, < .001
*non-White*	211 (20.23)^a^	1770 (35.71)^b^	
Job type			
*Faculty*	179 (17.16)	1010 (20.38)	5.61,.018
*Support Staff*	864 (82.84)	3946 (79.62)	
Age groups			
*18–40*	219 (20.99)	1813 (36.58)	178.46, < .001
*41–55*	584 (55.99)	1704 (34.38)	
*56+*	240 (23.01)	1439 (29.04)	
Education level			
*High School/GED*	81 (7.77)	N.A.	
*Some College No Degree*	235 (22.53)	N.A.	
*Associate degree*	124 (11.89)	N.A.	
*Bachelor Degree*	213 (20.42)	N.A.	
*Graduate Degree*	390 (37.39)	N.A.	
Years of service			
*Less Than a Year*	100 (9.58)	NA	
*1–3 years*	193 (18.50)	NA	
*3–5 years*	156 (14.95)	NA	
*5–10 years*	232 (22.24)	NA	
*10–15 years*	144 (13.81)	NA	
*5–20 years*	98 (9.39)	NA	
*20 years+*	120 (11.51)	N.A.	
Area of work			
*School of Medicine*	390 (37.39)	2151 (43.40)	94.19, < .001
*School of Nursing*	74 (7.09)	851 (17.17)	
*School of Health Professions*	68 (6.51)	207 (4.18)	
*School of Pharmacy*	61 (5.84)	237 (4.78)	
*Grad School of Biomedical Sciences*	21 (2.01)	27 (0.54)	
*Administration/Other*	429 (41.12)	1483 (29.92)^c^	

*Note*.

^a^, Since the response rate for those identifying as African American, American Indian, Asian, Hawaiian/Pacific Islander, multiracial, and "other" was too low, these categories were combined as "non-White" to yield an unbiased causal-comparative model.

^b^, Human Resources included Hispanics in this category.

^c^, Human Resources does not include administration as an area of work. The other category is comprised of the following work areas, research, rural and community health, external relations, managed health care, institutional compliance, finance, physical plant operations, human resources, president, information technology, and provost.

### Gender differences on the Organizational Civility Scales

Statistically significant differences across gender were found for *frequency of incivility* (*p* < .001). Female respondents (Median = 3.37, IQR = 3.1–3.9) experienced higher *frequency of incivility* followed by respondents who did not report/describe gender (Median = 3.23, IQR = 2.8–4.4) as compared to males (Median = 3.19, IQR = 2.8–3.6). Post-hoc testing revealed statistically significant differences between males and females (*p* < .001). Significant gender differences were found in the *perceptions of organizational climate* and *employee satisfaction*, with males viewing *organizational climate* as more positive (Median = 3.88, IQR = 3.3–4.4) compared to females (Median = 3.69, IQR = 2.9–4.1) and those who did not report or preferred not to describe (Median = 3.03, IQR = 2.6–3.7) (p < .001). Male respondents (Median = 76.33, IQR = 56.8–89.1) reported higher *employee satisfaction* than females (Median = 73.83, IQR = 52.0–87.0) and those who did not report/describe gender (Median = 52.92, IQR = 32.8–68.1; *p* = .002). Post-hoc test results with both subscales found statistically significant differences between females and males (*p* = .002) and those who did not report/describe gender and male respondents (*p* = .002). Those who chose not to report/describe gender reported a lower rating of overall civility (Median = 61.20, IQR = 42.3–76.0) when compared to females (Median = 74.20, IQR = 60.0–81.0) and males (Median = 75.90, IQR = 61.2–81.2) (*p* = .007). Post-hoc pairwise comparisons revealed that there is a statically significant difference between those who did not report/describe gender and females (*p* = .01), and those who did not report/describe gender and males (*p* = .005). No statistically significant difference was observed between males and females (see Table 3).

**Table 3:** Gender differences on the Organizational Civility Scale (OCS).

Subscale	Female *n* = 799		Male *n* = 216		Did Not Report/Describe Gender *n* = 28		P-value
	Mean (S.D.)	Median (IQR)	Mean (S.D.)	Median (IQR)	Mean (S.D.)	Median (IQR)	
Frequency of incivility	3.44 (0.64)	3.37 (3.1–3.9)	3.25 (0.62)	3.19 (2.8–3.6)	3.44 (0.87)	3.23 (2.8–4.4)	**< .001**
Overall Civility Rating	69.33 (17.73)	74.20 (60.0–81.0)	70.91 (15.90)	75.90 (61.2–81.2)	59.22 (19.42)	61.20 (42.3–76.0)	.**007**
Perceptions of Organizational Climate	3.54 (0.91)	3.69 (2.9–4.1)	3.78 (0.87)	3.88 (3.3–4.4)	3.18 (0.93)	3.03 (2.6–3.7)	**< .001**
Ratings of importance civility resources	3.15 (1.09)	3.11 (2.3–4.0)	3.41 (1.00)	3.28 (2.7–4.2)	2.64 (1.12)	2.39 (1.8–3.6)	**< .001**
Ratings of existence of civility resources	3.50 (0.99)	3.60 (3.0–4.3)	3.61 (0.98)	3.70 (3.0–4.4)	2.81 (1.13)	2.50 (2.1–3.8)	.**001**
Feeling about current Employment	3.80 (0.67)	3.75 (3.3–4.3)	3.82 (0.71)	3.67 (3.3–4.3)	3.99 (0.80)	4.00 (3.4–4.6)	.39
Employee Satisfaction	68.55 (22.66)	73.83 (52.2–87.0)	71.26 (21.18)	76.33 (56.8–89.1)	53.88 (24.17)	52.92 (32.8–68.1)	.**002**
Sources of Stress	3.12 (1.03)	3.20 (2.4–3.8)	3.00 (0.93)	3.00 (2.4–3.6)	3.34 (0.83)	3.20 (2.9–4.2)	.13
Coping Strategies	2.95 (0.45)	3.00 (2.7–3.2)	2.89 (0.46)	2.83 (2.7–3.2)	2.96 (0.43)	3.00 (2.7–3.2)	.15
Overall Levels of Stress	57.73 (26.20)	60.00 (40.0–80.0)	54.02 (26.73)	55.00 (33.3–75.0)	63.79 (27.41)	68.50 (46.3–83.8)	.**07**
Overall Levels of Coping Ability	40.17 (28.42)	32.00 (18.0–60.0)	37.73 (28.09)	31.50 (10.5–59.8)	39.64 (27.33)	50.00 (10.0–60.0)	.48

*Note*. SD = standard deviation; IQR = interquartile range; All the P-values were obtained from Independent-Samples Kruskal- Wallis test.

Respondents who did not report/describe gender (*n* = 28, 2.68%) gave the lowest ratings of the *existence of civility resources* (Median = 2.81, IQR = 2.10–3.80) in comparison to males (Median = 3.70, IQR = 3.00–4.40) and females (Median = 3.60, IQR = 3.00–4.30) (*p* = .001). Post-hoc test results with this subscale showed statistically significant differences between those who did not report/describe gender and females (*p* = .003) and those respondents who did not report/describe gender and males (*p* = .001). Additionally, respondents who did not report/describe gender gave the lowest scores to *importance of civility resources* (Median = 2.39, IQR = 1.80–3.60) when compared to male (Median = 3.28, IQR = 2.70–4.20) and female respondents (Median = 3.11, IQR = 2.30–4.00), (*p* < .001). Post-hoc test results with this subscale showed statistically significant differences between those who did not report/describe gender and females (*p* = .04), those who did not report/describe gender and males (*p* = .001), and between females and males (*p* = .10).

### Race differences on the Organizational Civility Scale

Statistically significant differences between racial groups (White vs. non-White) were found in the *existence of civility resources* (see Table 4). White respondents rated the existence of civility resources higher (Median = 3.65, IQR = 3.0–4.3) than non-White respondents (Median = 3.40, IQR = 2.6–4.2) (*p* = .048) (see Table 4). No significant differences were found on other subscales.

**Table 4:** Race differences on the Organizational Civility Scale (OCS).

Subscale	White *n* = 832		non-White n = 211		P-value
	Mean (S.D.)	Median (IQR)	Mean (S.D.)	Median (IQR)	
Frequency of incivility	3.40 (0.64)	3.31 (3.1–3.8)	3.38 (0.67)	3.29 (2.9–3.9)	.53
Overall civility rating	69.41 (17.23)	74.20 (60.4–80.4)	69.32 (18.54)	75.00 (58.0–82.0)	.60
Perceptions of Organizational climate	3.60 (0.90)	3.69 (3.0–4.3)	3.51 (0.94)	3.63 (2.8–4.1)	.22
Ratings of importance of civility resources	3.22 (1.06)	3.22 (2.4–4.0)	3.08 (1.15)	3.00 (2.2–3.9)	.10
Ratings of existence of civility resources	3.54 (0.97)	3.65 (3.0–4.3)	3.36 (1.09)	3.40 (2.6–4.2)	.**048**
Feeling about current employment	3.80 (0.68)	3.75 (3.25–4.25)	3.81 (0.67)	3.67 (3.3–4.3)	.81
Employee satisfaction	69.20 (22.07)	74.17 (53.3–87.5)	66.79 (24.28)	71.33 (45.8–86.7)	.30
Sources of stress	3.09 (1.01)	3.20 (2.4–3.8)	3.16 (0.99)	3.20 (2.6–3.8)	.42
Coping strategies	2.94 (0.44)	3.00 (2.7–3.2)	2.95 (0.50)	3.00 (2.7–3.2)	.68
Overall Levels of Stress	57.23 (25.88)	60.00 (40.0–80.0)	56.72 (28.36)	60.00 (35.0–80.0)	.99
Overall Levels of Coping Ability	39.20 (28.22)	31.00 (15.3–60.0)	41.43 (28.69)	44.00 (15.0–61.0)	.33

*Note*. SD = standard deviation; IQR = interquartile range; All the P-values were obtained from Independent- Samples Mann-Whitney U test.

### Faculty vs. staff differences on the Organizational Civility Scale

*Perceptions of organizational climate*, the *importance of civility resources*, *employee satisfaction*, *and overall stress level* were significantly different between the faculty and staff (see Table 5). *Perception of organizational climate* was higher among faculty respondents as compared to staff (Median = 3.94 vs 3.63, IQR = 3.3–4.4 vs 2.9–4.1, *p* = .001). The *importance of civility resources* rated higher among faculty than staff (Median = 3.33 vs 3.11, IQR = 2.7–4.2 vs 2.3–4.0, *p* = .02). Faculty had higher *employee satisfaction* than staff (Median = 79.17 vs 72.58, IQR = 58.3–89.3 vs 51.7–86.8, *p* = .01). Overall stress level was higher among faculty than staff (Median = 70.00 vs 60.00, IQR = 52.0–80.0 vs 38.3–80.0, *p* = .03).

**Table 5:** Job differences on the Organizational Civility Scale (OCS).

Subscale	Faculty *n* = 179		Staff *n* = 864		P- value
	Mean (S.D.)	Median (IQR)	Mean (S.D.)	Median (IQR)	
Frequency of incivility	3.33 (0.57)	3.26 (3.0–3.7)	3.41 (0.66)	3.34 (3.0–3.8)	.09
Overall civility rating	71.86 (15.91)	76.00 (64.4–81.0)	68.88 (17.77)	74.00 (59.1–80.8)	.09
Perceptions of Organizational climate	3.76 (0.93)	3.94 (3.3–4.4)	3.54 (0.90)	3.63 (2.9–4.1)	.**001**
Ratings of importance of civility resources	3.36 (1.06)	3.33 (2.7–4.2)	3.16 (1.08)	3.11 (2.3–4.0)	.**02**
Ratings of existence of civility resources	3.55 (1.02)	3.60 (3.0–4.4)	3.49 (0.99)	3.60 (2.9–4.3)	.47
Feeling about current employment	3.79 (0.74)	3.67 (3.3–4.2)	3.82 (0.67)	3.75 (3.3–4.3)	.25
Employee satisfaction	72.80 (20.33)	79.17 (58.33–89.33)	67.87 (22.90)	72.58 (51.7–86.8)	.**01**
Sources of stress	3.11 (1.02)	3.20 (2.4–3.8)	3.10 (1.00)	3.20 (2.4–3.8)	.93
Coping strategies	2.98 (0.41)	3.00 (2.67–3.17)	2.93 (0.46)	3.00 (2.7–3.2)	1.47
Overall Levels of Stress	60.96 (25.29)	70.00 (52.0–80.0)	56.33 (26.56)	60.00 (38.3–80.0)	.**03**
Overall Levels of Coping Ability	38.98 (27.91)	32.00 (16.0–60.0)	39.79 (28.42)	31.50 (15.0–60.0)	.79

*Note*. SD = standard deviation; IQR = interquartile range; All the P-values were obtained from Independent-Samples Mann-Whitney U test.

## Discussion

Incivilities represent a dangerous aspect of an organization’s culture, with the ability to impact individuals at numerous levels and undermine the organization’s mission itself. In healthcare and healthcare training facilities, creating and upholding policies regarding incivilities may be crucial to a healthy organizational system. However, to fully understand incivilities within such an organization, it is necessary to identify who is being impacted and how and to what (perceived) extent. Several implications are highlighted from this research.

A higher proportion of participating females (76.61%) in our study as compared to the population of female employees at the AHSC (71.79%) responded to the survey. Although females often participate in surveys at a higher rate than males, our results may indicate that females especially feel a desire to report (anonymously) on their organizational experiences [39]. Important to note is that different genders may have varying perceptions of the organizational climate as well as the availability of resources. Gender played a role in respondents reporting on organizational climate, with males viewing it slightly more positively, reporting slightly higher beliefs in the existence of civility resources, slightly higher overall workplace civility, and increased employee satisfaction. In terms of reported frequency of incivilities, females reported slightly higher frequencies than males, which is similar to previous research [5, 7, 17, 40]. In our study, those who did not report/describe gender reported the same average frequency of incivilities as females. Females and those who did not report/describe gender, when compared to males, perceived the existence of fewer available civility-upholding resources and had a lower view of the importance of such resources. Because those who assigned the least importance to civility resources were also those who felt there were the fewest resources (i.e., those who did not report gender), it is essential to remember that employees cannot know the benefits of civility resources if they are unaware of those resources. Therefore, making the resources more apparent and easily accessible is of grave importance. Our results emphasize the need to communicate about resources more clearly to all employees, regardless of any uncertainties in who might "need" the information most, and to identify ways for everyone to feel comfortable reporting uncivil behavior, irrespective of their sex or gender.

Our findings also speak to considerations of race when developing and rolling out organizational resources related to civility. In our research, White respondents perceived the existence of civility resources to be slightly higher. Our research does not support Cortina and colleagues’ [7] finding that people of color experienced significantly higher rates of job incivility than White individuals. We found no significant differences between race and frequency of incivility. The Cortina et al. [7] study included law enforcement, city government, and the U.S. military, which could potentially carry different racial stereotypes in the job when compared to healthcare training. Asfaw and colleagues’ [5] study produced no significant differences in workplace mistreatment based on race. Therefore, race-based differences may be a function of the particular work industry or other complex organizational issues (i.e., positional power, power dynamics) beyond the scope of this study. However, there was a significant difference in non-White respondents (20.23%) in our study as compared to the population of non-White AHSC employees (35.71%). The finding may be based on concerns of confidentiality expressed to our researcher team before and during the survey period; we cannot assume all employees felt safe enough to participate (i.e., fear of retribution from their supervisor) despite researcher assurances.

We also examined type of work. The faculty in our research reported a slightly more positive outlook of the organizational climate and rated civility resources as slightly more important than staff. Based on their perceptions of organizational climate, it is not surprising that faculty also reported more satisfaction, despite their higher reports of overall stress compared to staff. In their concept analysis review of previous nursing literature, Liu, Aungsuroch, and Yumibhand [41] found that research had identified a decrease in nurses’ burnout, absenteeism, work stress, and intentions to quit when they were satisfied with their jobs, and that their job satisfaction affected patient satisfaction and quality care. Therefore, it is important that leadership works toward increasing job satisfaction for all employees and focus efforts on ways to do so for staff in particular.

### Study limitations and future directions

Despite the contributions made by this study, there are several limitations to consider. "Hispanic" or "Latino" was not included as a specific race/ethnicity category in the survey, and more research is needed on nuanced differences beyond White and non-White employees. The current study was based on only one AHSC, limiting generalization. Several respondents were concerned about reporting on incivilities in the workplace, reaching out to the research team to receive further assurance of the confidentiality of their responses. This reaction points to the fears and anxieties related to incivility and potential retribution. Although the researchers attempted by standard means to make it clear in the recruiting emails, some employees likely chose not to participate due to concern over being identified. In a similar vein, some employees may have chosen not to respond if they felt satisfied with their jobs or did not feel there were civility issues to report. Based on the sample’s voluntary nature, there is a potential bias in the results based on who selected to participate. Future research should consider additional means of communicating confidentiality to respondents as well as how to better communicate the research value of their participation regardless of job satisfaction levels.

Our study artificially limited the possible perceived effects of incivility in the workplace to the actual healthcare workers themselves. We did not study the "customers" or "clients"–that is, patients, families, loved ones, or anyone who could be affected by interaction with health professionals, but our research provides a springboard for conducting such incivility scholarship in healthcare settings. Indeed, this is an understudied area of incivility in the health workplace that demands further investigation. It is not an unreasonable hypothesis to posit that higher stress levels of frontline "high touch" providers like the nursing staff may lower the quality of the care for the patient. In other words, organizational health may affect patient health. Satisfaction among healthcare professionals may be an intervening variable in health outcomes. Alternately, a counter-hypothesis may be that at moderate or even higher stress levels, employee training and professional codes override personal feelings, so that the state of patient care remains acceptable.

Finally, we did not study whether there might be different effects within different groups, areas, or units within the AHSC system. Are there parts of an AHSC where there are nodes of civility or instability, areas where there are higher rates of worker unhappiness or happiness? What might affect their existence? No large institution operates within a homogeneous workplace culture nor, as we have seen, uniform perfection of civility. An institution that may have an overall highly civil culture may contain groups or units that are suffering from a toxic culture; the opposite situation may exist as well. Therefore, future civility instrument development and research should capture participants’ departmental affiliation, and their subunits within schools, departments, and administrative units with more detail, so civility practices can be examined in depth (through utilizing multivariate models such as hierarchical linear models [HLM]) to capture the nested nature of organizational civility practices and resources within the schools, departments, programs, and units in the same institution.

Findings from this research could be useful to institutions desiring to combat incivilities in the healthcare workplace and educational environments. Dyrbye et al. [26] posited the need for researchers to identify healthcare system and organizational factors leading to burnout for healthcare providers. This study provides a necessary starting point for scholars to further explore AHSCs in terms of civility policies and trainings.

## Conclusions

Our study aimed to characterize incivility at an AHSC experienced by faculty and staff and to understand gender, racial, and differences by employee category among a range of incivility-associated factors. We found that men viewed organizational climate somewhat more positively along with a slightly higher report of overall workplace civility, reported a somewhat higher existence of and importance of civility resources, and experienced increased employee satisfaction. Females and those who did not report/describe gender reported slightly higher frequencies of incivility. White respondents perceived the existence of civility resources to be slightly higher. Faculty reported a somewhat more favorable opinion of organizational climate, rated civility resources as a bit more important, and reported higher satisfaction than staff, despite faculty’s higher reports of stress. Limitations of our study include self-selection bias, a homogenous sample, and findings cannot be generalized beyond the study population. Our research adds to existing incivility research that examines a range of workplace settings and employee characteristics. We anticipate that our results will inform civility training, policy and procedures, and the implementation of other evidenced-based strategies, thereby shifting the culture of health science centers to a more civil climate and improving employee satisfaction and well-being.

## Supporting information

S1 Dataset (XLSX)
